# Novel HSPB8 mutations in severe early-onset myopathy with involvement of respiratory and cardiac muscles cause proteostasis defects in cell models

**DOI:** 10.1038/s41431-025-01868-z

**Published:** 2025-06-04

**Authors:** Barbara Tedesco, Stojan Peric, Goknur Selen Kocak, Jiayan Tan, Han Duong, Ana Töpf, Vidosava Rakocevic-Stojanovic, Sanja Milenkovic, Yolande Parkhurst, Liliane Gibbs, Angela Martin-Rios, Pier D. Lambiase, Oliver P. Guttmann, Chiara Marini-Bettolo, Elizabeth Harris, Matthew B. Harms, Vukan Ivanovic, Veronica Marchesi, Margherita Milone, Vincent Timmerman, Volker Straub, Angelo Poletti, Virginia Kimonis

**Affiliations:** 1 Laboratory of Experimental Biology, Dipartimento di Scienze Farmacologiche e Biomolecolari “Rodolfo Paoletti” (DiSFeB), Dipartimento di Eccellenza 2018–2027, Università degli Studi di Milano, Milan, Italy; 2 University of Belgrade, Faculty of Medicine, University Clinical Center of Serbia - Neurology Clinic, Belgrade, Serbia; 3 John Walton Muscular Dystrophy Research Centre, Institute of Translational and Clinical Research, Newcastle University and Newcastle Hospitals NHS Foundation Trust, International Centre for Life, Newcastle upon Tyne, UK; 4 Department of Pediatrics, University of California Irvine, Irvine, CA USA; 5 Western University of Health Sciences, College of Osteopathic Medicine of the Pacific, Pomona, CA USA; 6 National Laboratory for Neuromuscular Biopsies, Clinical Hospital Center Zemun, Belgrade, Serbia; 7 Muscle Immunoanalysis Unit, Newcastle upon Tyne Hospitals NHS Foundation Trust, Newcastle upon Tyne, UK; 8 Pediatric Radiology, Department of Radiology, University of California Irvine, Irvine, CA USA; 9 University College London, Barts Heart Centre, London, UK; 10 Department of Neurology, Columbia University Medical Center, New York, NY USA; 11 Department of Neurology, Mayo Clinic, Rochester, MN USA; 12 Peripheral Neuropathy Research Group, Department of Biomedical Sciences and Institute Born Bunge, University of Antwerp, Antwerpen, Belgium

**Keywords:** Disease genetics, Medical genetics

## Abstract

Heat shock protein family B (small) member 8 (HSPB8) promotes chaperone-assisted selective autophagy (CASA), which assures proteostasis in muscles and neurons. *HSPB8* frameshift mutations found in neuromyopathies are translated on the same frame, generating the same C-terminal extension, which causes HSPB8 aggregation and proteostasis defects. Here, we describe three novel *HSPB8* frameshift variants, translated to protein using the third alternative frame to stop codons downstream to the canonical one and to the one used by other known *HSPB8* frameshift mutants. Therefore, these variants are predicted to encode a C-terminal extension that is different in length and amino acids. *HSPB8* c.562delC and c.520_523delTACT were identified in two unrelated sporadic patients, while c.515delC, in a familial case of early-onset myopathy. Patients may differentially exhibit additional pathological features, such as neuropathy, respiratory insufficiency, and, remarkably, severe cardiomyopathy. Skeletal muscle biopsies revealed variations in fiber size, atrophy, multiple vacuoles, fat infiltration, and eosinophilic inclusions. In a reconstituted cell model of disease the expression of one representative novel HSPB8 mutant results in i) aggregation of the HSPB8 mutant, ii) sequestration of both the HSPB8 wild-type and CASA complex members, as well as iii) the autophagy receptor sequestosome-1 (SQSTM1/p62), iv) accumulation of ubiquitinated substrates, and v) defects in CASA-mediated degradation. Our results prove that the last exon of the *HSPB8* gene is highly susceptible to pathogenic mutations, resulting in a wider phenotypic spectrum associated with *HSPB8* frameshift variants. Our studies suggest the importance of *HSPB8* genetic testing not only for neuropathy and myopathy but also for cardiomyopathy.

## Introduction

Chaperone heat shock protein (HSP) family B member 8 (HSPB8) is highly expressed in muscles and neurons, where it promotes the degradation of potentially harmful proteins through chaperone-assisted selective autophagy (CASA) [1, 2]. At a molecular level, HSPB8 facilitates autophagy by interacting with BAG cochaperone 3 (BAG3), an HSP family member A (HSPA), and the E3 ubiquitin ligase STUB1 (STIP1 homology and U-box containing protein 1), forming the CASA complex [3]. Client substrates recognised and ubiquitinated by the CASA complex recruit the autophagy receptor sequestosome-1 (SQSTM1/p62), which links them to MAP1LC3 (microtubule-associated protein 1A/1B-light chain 3, or LC3) exposed on forming autophagosomes. In skeletal and cardiac muscles, CASA maintains Z-disks upon mechanical tension; in neurons, CASA promotes clearance of aggregating proteins causative of neurodegeneration [1, 4–7].

Missense mutations in *HSPB8* cause Charcot-Marie-Tooth type 2L neuropathy, distal hereditary motor neuropathy type 2A, and, rarely, distal myopathy [8–11]. In contrast, frameshift *HSPB8* mutations cause a unique myopathy with myofibrillar aggregates and rimmed vacuoles, with or without distal motor neuropathy (Supplementary Table 1) [9, 12–15]. We characterised *HSPB8* frameshift mutations encoding HSPB8 sharing the same abnormal C-terminal extension, a highly hydrophobic region causing HSPB8 aggregation, CASA complex member sequestration, and proteotoxicity [16].

Herein, we extend the mutational and phenotypic spectrum of HSPB8-related neuromyopathies, identifying three new *HSPB8* frameshift variants (c.562delC, c.520_523delTACT, and c.515delC) causing neuromyopathy with severe respiratory and cardiac involvement. We showed here that the new *HSPB8* variants use the third open reading frame (ORF) after mutations, producing HSPB8 (p.Q188Rfs*59, p.Y174Qfs*72, and p.P172Lfs*75, respectively) with a completely different C-terminal extension compared to those previously reported, but still causing HSPB8 aggregation and proteostasis alterations, suggesting a common pathogenic mechanism.

## Subject and methods

### Subject evaluation

Patients' recruitment (in Serbia, UK, and USA) and ethical approval are described in Supplementary Materials. All patients underwent thorough neurological examination, nerve conduction study (NCS), and needle electromyography (EMG). Muscle strength was scored using the Medical Research Council (MRC) Muscle Grading Scale [17]. All underwent spirometry, electrocardiogram (ECG), echocardiography, and muscle magnetic resonance imaging (MRI) with T1-weighted (T1w), T2-weighted (T2w), and 3-point Dixon [18, 19]. Whole exome sequencing (WES) was performed: patient I at the Broad Institute of Harvard and MIT (Cambridge, MA, USA) as part of the international MYO-SEQ project [20]; patient II at Newcastle NHS Trust Hospital, UK; patient III at University of California Irvine, USA, with trio genome sequencing on the proband, his affected mother, and unaffected father.

### Histology and immunohistochemistry (IHC)

Muscle biopsies were analysed by optical microscopy, as described [21]. Tissue fragments frozen in liquid-nitrogen-cooled isopentane were cut on a cryotome at 8 or 10 µm. Cryosections were stained with H&E and modified Gomori trichrome. IHC analyses of patient I were performed on 10% formalin-fixed tissue fragments embedded in paraffin. Four-micrometer sections were stained with antibodies listed in Supplementary Table 2. ThermoScientific CryoStar NX70 automated immunostainer with 3,3′-diaminobenzidine detection was used (ThermoFisherScientific, Waltham, MA, USA), and sections were imaged with Leica DM2000 microscope with digital imaging camera (Leica DFC295) and software LAS V4.7. For IHC analyses on patient II, the tissue was frozen in cooled isopentane, sectioned (6 μm), and permeabilised in Tris-buffered saline (TBS) (0.1% Triton X-100 in Phosphate Buffered Saline (PBS)) at room temperature, and incubated in primary antibody (Supplementary Table 2) overnight at 4 °C. After TBS washing, secondary antibodies were applied and labeling developed with Betazoid DAB chromogen (CellPath, Newtown, Powys, UK). Nuclei were counterstained with Carazzi’s haematoxylin.

### In silico and in vitro studies

Camsol analyses are described in Supplementary Materials. Neuroblastoma X Spinal Cord 34 (NSC34) cells were cultured, transfected, and analysed as reported in Supplementary Material as previously described [16].

## Results

### Clinical presentation and examination findings

Patient I, a Serbian male, noticed symptoms before 20 years of age, with weakness of distal legs that progressively affected proximal leg muscles. Five years later, he was only walking with a walker. Before 30 years of age, he showed a high arched palate, severe atrophy and weakness of the sternocleidomastoid muscles, moderate weakness of the *trapezius* muscles, limited neck flexion (rigid spine syndrome), modest atrophy of the shoulder muscles with mild scapular winging on the right side, and mild wasting of the forearm muscles. Muscle strength was reduced in all shoulder muscles (MRC 4), the finger flexor (MRC 4), and small hand muscles (MRC 3). He showed moderate atrophy of the pelvic girdle and proximal leg muscles, and severe atrophy of all lower leg compartments. Muscle strength was severely reduced symmetrically, with the hip abductors showing MRC 3, other proximal muscles 2, foot dorsiflexors 1 with severe foot drop and ankle contractures, and feet plantar flexors 3, with a waddling gait with prominent foot drop. Severe weakness of trunk muscles was noted, with difficulty in raising from lying to sitting positions, and requiring assistance to rise from a sitting position. Reflexes were reduced in his arms and absent in his legs. Sensation was normal in his extremities. The patient was born with a reduced weight for gestational age (1800 g) and started to walk at 20 months of age. Serum creatine kinase (CK) activity was elevated up to 550 IU/L (male reference value up to 170 IU/L). Patient’s parents and sister had normal neurological findings and CK levels.

Patient II is a British woman in her 30s with history of progressive muscular and cardiac issues. Symptoms began in her first decade of life, with physical limitations, including inability to perform sit-ups, and developed mild scoliosis. Muscle weakness predominantly affected her truncal, abdominal, and proximal lower limb muscles, significantly impairing her ability to sit up, although she denies any weakness in her upper limbs. During her first years of life, she was diagnosed with Kawasaki syndrome, followed by cardiomyopathy. Over time, her condition progressed to restrictive hypertrophic cardiomyopathy requiring implantation of a cardioverter-defibrillator for ongoing management, regular follow-up by cardiology teams. Next, she developed increasing shortness of breath, which gradually worsened. On examination, cranial nerves were intact. A mild proximal upper limb weakness with bilateral scapular winging and significant truncal weakness, with proximal weakness of pelvic girdle muscles (MRC 3), impacted her ability to rise from a lying position and from a chair. Bilateral foot drop was noted, attributed to weakness in the ankle dorsiflexors (MRC 3). The patient has a stiff spine with mild scoliosis. She walks independently with a single walking stick, managing ~100 m before requiring a rest, but with joint pain in the hips during walking. Sensory examination was normal, and both heart and lung sounds were normal on auscultation. The patient has no known family history of similar conditions. Socially, she is an ex-smoker, consumes alcohol socially, and has no history of illicit drug use.

Patient III is an American Caucasian male of Ashkenazi Jewish descent in his late 30s. He reported difficulty running from early childhood, attributed to flat feet not improved by a series of orthotics. By the end of high school, he was no longer able to run, with difficulties in climbing stairs in his 20s, when he began seeing neurologists. Muscular dystrophy was suspected. CK was chronically elevated (~400 IU/L). Sensory nerve conduction studies were normal. Muscle and nerve biopsy showed a vacuolar myopathy. Two extensive next-generation sequencing panel tests identified six candidate variants of uncertain significance. He sought additional work-up through the undiagnosed neuromuscular disease program at Mount Sinai Medical Center, New York, USA. On evaluation in his 30s, examination showed normal facial strength, but asymmetric scapular winging, scoliosis, and a positive Beevor’s sign, suggesting axial muscle weakness. Strength testing examination of the arms revealed weakness of his shoulder abductors (MRC 4 right/5 left). The left hip flexors and ankle dorsiflexors were MRC 4; all other muscle groups were normal. Sensory examination and reflexes were normal. He had excessive lordosis and increased pelvic tilt while walking.

### Electrodiagnostic and Muscle MRI

**Fig. 1:**
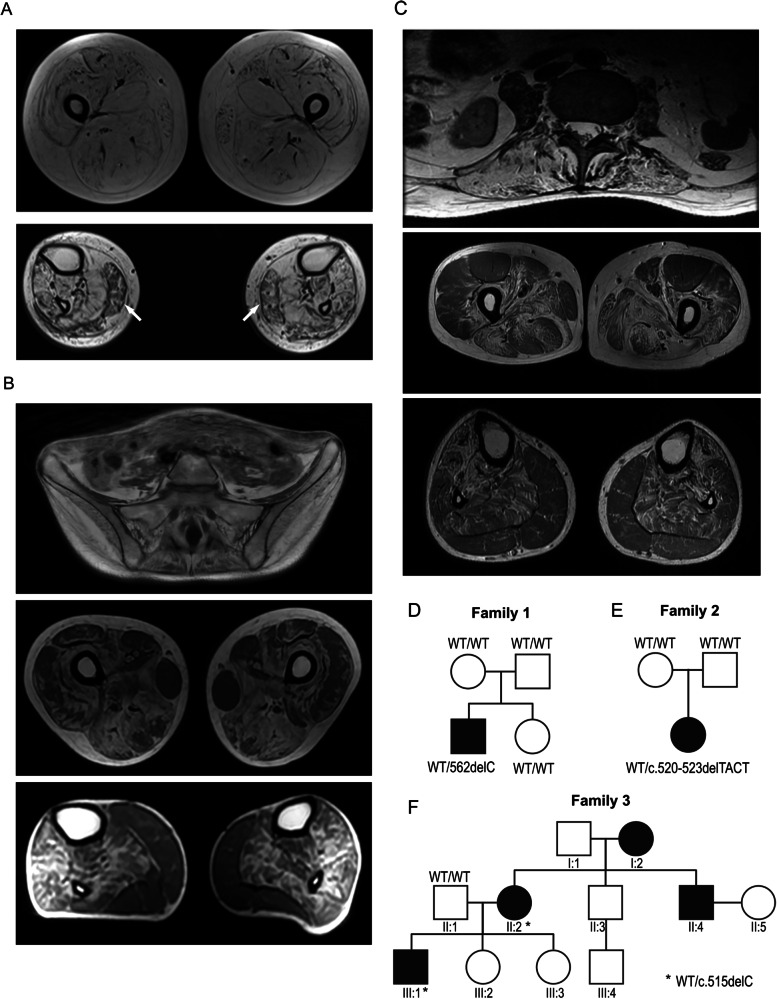
Muscle MRI and pedigree. **A** Patient I muscle MRI of the lower extremities performed before 30 years of age showing severe fatty replacement of all proximal muscles (top) and severe involvement of distal muscles with relative sparing of the *gastrocnemius* (arrowed, bottom). **B** Patient II muscle MRI performed in her early 30s shows extensive fat replacement in the spinal and *gluteus* muscles, with sparing of the *iliacus* muscle in the pelvis (top); severe fatty replacement affecting the posterior muscle group, with significant involvement in the *vastus intermedius* and *vastus medialis*; the *vastus lateralis* shows milder involvement, while the *rectus femoris*, *gracilis*, and *sartorius* muscles remain relatively spared, as observed in the thigh (middle); severe fatty replacement, with relative sparing of the *gastrocnemius* muscles, both *lateralis* and *medialis*, is observed in the lower leg (bottom). **C** Patient III muscle MRI performed in his 30s shows paraspinal muscle fatty infiltration and *psoas* muscle atrophy (top); marked hamstring atrophy with relative sparing of the *rectus femoris*, *vastus lateralis*, *gracilis*, and *sartorius* muscles in the thigh (middle); fatty infiltration and atrophy with relative sparing of *gastrocnemius* and *peroneus* muscles in the calf (bottom). **D** Patient I family pedigree of HSPB8 c.562delC de novo mutation. **E** Patient II family pedigree of HSPB8 c.520-523delTACT de novo mutation. **F** Patient III family pedigree of HSPB8 c.515delC mutation showing autosomal dominant inheritance. The filled squares and circles indicate affected males and females with a history of muscular weakness. Asterisks indicate genetic testing results of HSPB8 c.515delC. Family members I:2 and II:4 could not be genotyped.

Patient I, in his 20s, underwent needle EMG that showed neurogenic involvement in the lower legs. Between 25 and 30 years of age, electrophysiology studies showed diffuse myopathic patterns with severe denervation in the lower legs; in his 30s, muscle MRI of the lower extremities revealed extensive fat replacement in all thigh muscles, each scoring 4 on the Mercuri scale, and moderate to severe fat replacement pattern in the lower leg muscles, scoring 3, except for the *soleus* muscle, which also scored 4 (Fig. 1A) [22].

The nerve conduction studies of patient II showed normal sensory and motor responses in both the upper and lower extremities, with normal F-wave responses. EMG revealed short-duration, mild polyphasic motor unit potentials in several muscles, including the right *serratus anterior*, *infraspinatus*, *supraspinatus*, and *serratus anterior*, as well as the left deltoid. Similar findings were noted in both lower extremities, specifically in the right and left *tibialis anterior*, *gastrocnemius*, and *rectus femoris* muscles. Her muscle MRI revealed significant fatty replacement in the paraspinal and *gluteus* muscles, with a Mercuri score of 3–4 (Fig. 1B) [22]; severe fatty replacement was observed in the thighs in the posterior muscle group, also scoring 3–4. The *vastus lateralis* showed less involvement, whereas the *vastus intermedius* and *vastus medialis* were significantly affected. In contrast, the *rectus femoris*, *gracilis*, and *sartorius* muscles remained relatively spared. In the lower legs, most muscles exhibited severe fatty replacement, scoring 3–4, while the *gastrocnemius* muscles, both *lateralis* and *medialis*, were relatively spared (Fig. 1B).

The nerve conduction studies of patient III showed reduced nerve output in the fibular nerve but otherwise normal motor and sensory responses in both upper and lower extremities. EMG revealed rapid recruitment of short, low-amplitude motor unit potentials with fibrillation potentials in axial (thoracic paraspinals, *infraspinatus*), proximal (*gluteus medius*, *biceps femoris*, *vastus medialis*), and distal (*tibialis anterior*) muscles. Lumbar MRI performed in his 30s showed severe thoracic paraspinal muscle, *psoas*, and gluteal muscle atrophy (Fig. 1C). The patient III thigh MRIs showed fat infiltration of most lower extremity muscles with marked and symmetric atrophy of bilateral thigh muscles but mild relative sparing of *rectus femoris* and *vastus lateralis*. The calf MRI showed moderate muscle atrophy with relative sparing of medial and lateral heads of *gastrocnemius* (Fig. 1C).

### Cardiac and respiratory system investigations

Patient I exhibited severe pulmonary restriction (forced expiratory volume (FVC) < 50%) before reaching his 30s. Echocardiography revealed severe hypertrophic cardiomyopathy, with a left ventricular wall thickness of up to 2.2 cm, preserved systolic function, impaired diastolic function, and no obstruction of the aortic outflow tract, with no documented cardiac conduction defects or arrhythmias. In his early 30s, he developed severe heart failure symptoms, including dyspnea, leg swelling, and fatigue. Despite medical treatment, he experienced respiratory arrest followed by cardiac arrest and passed away in his 30s. Cardiac MRI was not performed.

Patient II exhibited severe pulmonary restriction, with an FVC of 50% of predicted and forced expiratory volume (FEV_1_) of 57% of predicted. Diagnosed with Kawasaki syndrome at age 3 years, she developed hypertrophic cardiomyopathy by age 4 years. Cardiac MRI, performed in her mid-20s, revealed significant left ventricular (LV) hypertrophy (maximal wall thickness 2.5 cm), subendocardial perfusion defects, and patchy fibrosis in the apical and septal regions. LV function was preserved (LV ejection fraction (EF) 65%), with mid-to-apical systolic obliteration but no systolic anterior motion (SAM) or LV outflow tract (LVOT) turbulence. A follow-up echocardiogram in her 30s confirmed moderate to severe concentric LV hypertrophy (maximal thickness 1.5 cm at the basal inferolateral wall), preserved systolic function (EF > 55%), and normal right ventricular size and function.

Patient III had pulmonary function testing in his 30s, showing a restrictive pattern: FVC of 69%, FEV1 of 68%, and total lung capacity (TLC) of 72%. Cardiovascular assessment, ECG, echocardiogram and 24-h ECG Holter monitoring revealed no abnormalities. Cardiac MRI indicated normal function, anatomy, and a clinically non-relevant pericardial effusion with normal pericardial thickness. Over the last seven years, strength declined in many muscles, and FVC worsened to 47% of predicted. His chest X-ray showed elevation of the left diaphragm from eventration thinning, indicating hemidiaphragm paralysis. Bilevel positive airway pressure (BiPAP) via face mask was recommended for his respiratory insufficiency.

### Genetic Investigation

Given their clinical presentation, the selection of potential disease-causing variants in patients I and II from the WES, and patient III from whole genome sequencing (WGS) analysis was first restricted to genes associated with a) neuropathies and neuromuscular disorders, and b) cardiomyopathies. The latter provided no potential candidate changes, the former identified heterozygous frameshift variants in the *HSPB8* gene: c.562delC; p.Q188Rfs*59 in patient I (Supplementary Fig. 1A), c.520_523delTACT; p.Y174Qfs*72 in patient II (Supplementary Fig. 1B), and c.515delC; P172Lfs*75 in patient III (Supplementary Fig. 1C). All variants are in the last exon and not present in the control population of over 120,000 exomes [http://gnomad.broadinstitute.org/faq]. Sanger sequencing of family members of patients I and II revealed the de novo status of both *HSPB8* frameshift variants, not present in any unaffected parents and siblings (Fig. 1D, E). Paternity was confirmed through exome data in both families. WGS trio performed in family 3 revealed the c.515delC variant in both patient III (indicated as III:1) and his affected mother. No DNA of the deceased affected uncle was available to confirm segregation.

All these variants are classified as pathogenic according to the ACMG (American College of Medical Genetics and Genomics) variant criteria: PVS1, PS2, and PM2 [23].

### Histopathology

**Fig. 2:**
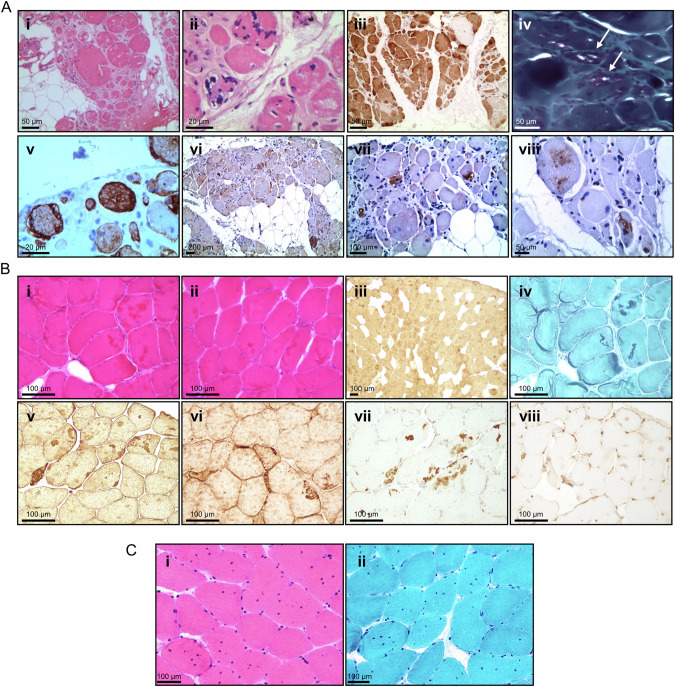
Histopathological analyses. **A** Histopathological findings of deltoid muscle collected before 30 years of age from patient I: (**i** and **ii**) H&E staining showing variation in fiber size, fatty replacement, fiber splitting, endomysial fibrosis, presence of vacuoles and multiple internal nuclei. (**iii**) Myosin fast staining of type 2 fibers. (**iv**) Gomori Trichrome shows the rimmed vacuoles containing fuchsinophilic material in certain fibers (arrows); (**v**) desmin staining revealing aggregation; (**vi**) HSPB8 is diffusely increased in atrophic fibers, (**vii**) SQSTM1/p62 is positive in rimmed vacuoles, and (**viii**) strong TDP-43 reactivity is detected in myofibrillar aggregates. **B** Histopathological findings of quadriceps muscle from patient II: (**i** and **ii**) H&E staining displaying variation in fiber size, atrophy, fat infiltration, focal fibrosis, eosinophilic inclusions, and rimmed vacuoles. (**iii**) Myosin fast staining of type 2 fibers; (**iv**) Gomori Trichrome and (**v**) desmin labeling revealing aggregation; (**vi**) HSPB8 cytoplasmic aggregates in vacuolated and atrophic fibers, (**vii**) SQSTM1/p62 labeling showing aggregation, and (**viii**) punctate TDP-43 labeling in vacuolated fibers. **C** H&E (**i**) and Gomori Trichrome (**ii**) staining of patient III left quadriceps muscle in his 30s showing increased fiber size variation, internal nuclei, and myofibrillar deposits.

Histopathological findings of deltoid muscle from patient I biopsied in his 30s showed a dystrophic pattern, variation in fiber size with marked atrophy and hypertrophy, significant fatty replacement, fiber splitting, multiple internal nuclei, endomysial fibrosis, and irregular myofibrillar organization with the presence of rimmed vacuoles and inclusions (Fig. 2A**i, ii**). Myosin fast staining showed predominance of type II fibers (Fig. 2A**iii**). Gomori Trichrome highlighted rimmed vacuoles (Fig. 2A**iv**), and desmin showed variable staining secondary to the pathology (Fig. 2A**v**). HSPB8 was diffusely increased in atrophic fibers (Fig. 2A**vi**). Rimmed vacuoles were positive for SQSTM1/p62 (Fig. 2A**vii**), while strong TDP-43 reactivity in myofibrillar aggregates indicated TAR DNA-binding protein (TDP)-43 pathology (Fig. 2A**viii**).

Patient II muscle biopsy (*quadriceps*) showed variation in fiber size, atrophy (some grouped), mild fat infiltration, focal fibrosis and inflammation, multiple rimmed vacuoles mainly present in angulated basophilic fibers, and occasional eosinophilic inclusions. Occasional elongated and angulated fibers were present, along with rare necrotic fibers (Fig. 2B**i, ii**). An overall predominance of type II fibers was noted (Fig. 2B**iii),** and basophilic aggregates were present on Gomori Trichrome (Fig. 2B**iv**). Strong desmin labeling of cytoplasmic aggregates was seen (Fig. 2B**v**). Defined HSPB8 labeling was observed in cytoplasmic aggregates, with diffuse cytoplasmic labeling present in vacuolated and atrophic fibers (Fig. 2B**vi**). Occasional SQSTM1/p62 labeling (Fig. 2B**vii**) coincided with areas of myotilin and desmin positivity and was also observed in rare peripheral vacuoles. Punctate TDP-43 was present in occasional vacuolated fibers (Fig. 2B**viii**).

Muscle biopsy of the left *quadriceps* of patient III revealed increased internalised nuclei and exceedingly rare rimmed vacuoles (Fig. 2C**i**). In trichrome-stained sections, several fibers showed hyaline inclusions (Fig. 2C**ii**), with a minimal collection of inflammatory cells at a perivascular site. A muscle biopsy of his mother revealed myofibrillar disruption, and a muscle biopsy of his uncle at 51 years of age showed features of active and chronic myopathy with a few rimmed vacuoles.

### In silico and in vitro analyses of the *HSPB8* frameshift variants

**Fig. 3:**
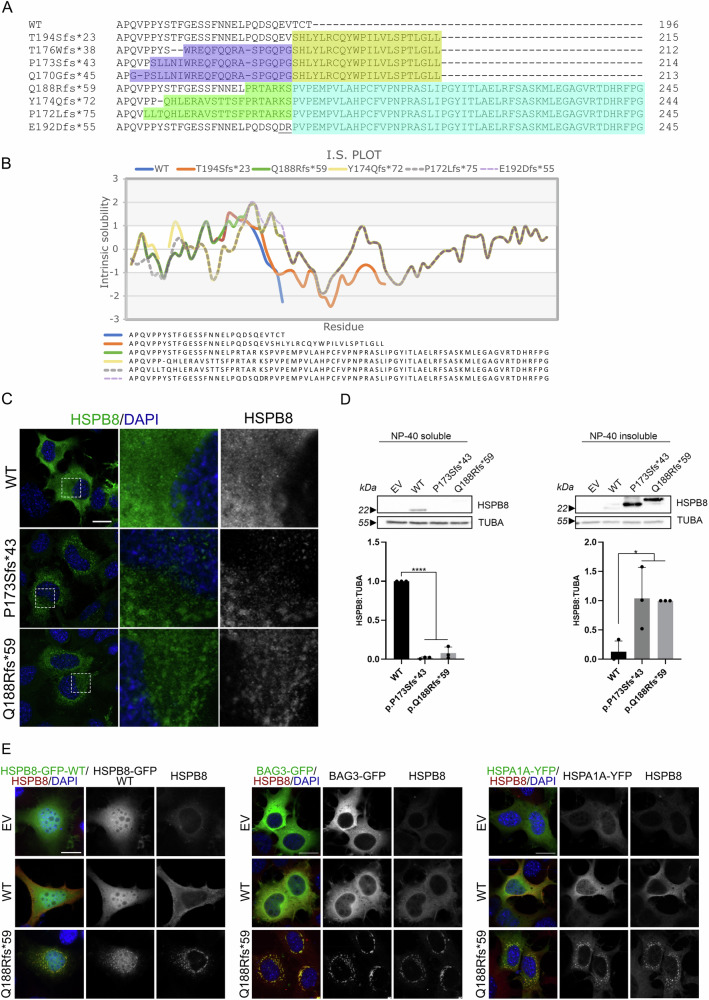
In silico and in vitro analyses of the novel HSPB8 variant C-termini. **A** Comparison of the C-terminus of the novel HSPB8 frameshift variants with the HSPB8 WT and the frameshift variants reported in the literature. The common C-terminal extension and variable C-terminal domain of the HSPB8 frameshift variants previously described are in yellow and purple, respectively [16]; the C-terminal extension and C-terminal modification of the novel HSPB8 frameshift variants p.Q188Rfs*59, p.Y174Qfs*72 and p.P172Lfs*75 (here reported) and E192Dfs*55 (reported in [24]) are in blue and green, respectively. The underlined amino acids for E192Dfs*55 are not present in any other sequence. **B** Intrinsic solubility plot of the C-termini of HSPB8 WT and the frameshift variants obtained with CamSol method [25]. **C** NSC34 cells expressing HSPB8 WT or its mutants p.P173Sfs*43 and p.Q188Rfs*59. HSPB8 is in green, and nuclei are stained with DAPI (blue); scale bar = 20 µm. **D** Western blot on NP-40 soluble and insoluble protein fractions of NSC34 transiently transfected with an empty vector (EV), HSPB8 WT or its mutant p.Q188Rfs*59 and p.P173Sfs*43 constructs. Bar graphs report the optical density relative to HSPB8 normalised on the soluble tubulin (TUBA). One-way ANOVA with Tukey’s test was performed (*p < 0.05; ****p < 0.0001); *n* = 3. HSPB8 was detected using an antibody recognizing the N-terminal region (NTR). **E** Immunofluorescence on NSC34 transiently transfected with an empty vector (EV), HSPB8 WT or p.Q188Rfs*59 (red) and HSPB8-WT-GFP (left, green), GFP-BAG3 WT (middle, green) or YFP-HSPA1A (right, green). Nuclei are stained with DAPI (blue); scale bar = 20 µm.

*HSPB8* frameshift mutations previously characterised [9, 12–15] are all predicted to encode HSPB8 proteins with variably different C-terminal regions, but identical C-terminal extension, being all shifted on the same ORF. This C-terminal extension spans ~20 amino acids, most highly hydrophobic. The novel *HSPB8* variants described here encode an elongated protein with an entirely different C-terminal extension from that previously identified, but identical to that of the variant c.576_579delinsCAG (p.E192Dfs*55) recently identified in a patient affected by early-onset myopathy [24] (Fig. 3A). The novel C-terminal extension is ~50 amino acids in length with both highly hydrophobic and highly hydrophilic amino acids [25], thus showing a variable intrinsic solubility (Fig. 3B).

Based on the different features of the two C-termini, we performed in vitro studies to compare their behaviours, since the previously reported exhibited high propensity to aggregation [16]. Thus, we chose HSPB8 p.Q188Rfs*59 as representative of the new variants, and compared it to HSPB8 WT and p.P173Sfs*43 by expressing them in motoneuronal NSC34 cells. As shown in Fig. 3C, both HSPB8 p.Q188Rfs*59 and HSPB8 p.P173Sfs*43 formed cytoplasmic aggregates, displaying very similar behaviour, while HSPB8 WT remained diffuse. NP-40 soluble/insoluble extraction of cell lysates revealed that both HSPB8 frameshift mutants (p.Q188Rfs*59 and p.P173Sfs*43) partitioned in the insoluble fraction (Fig. 3D). The HSPB8 p.Q188Rfs*59 displayed a decreased electrophoretic mobility compared to HSPB8 WT and p.P173Sfs*43 mutant, in line with the predicted molecular weight of the encoded proteins.

HSPB8 forms the CASA complex together with BAG3 and HSPAs. Thus, we tested if HSPB8 p.Q188Rfs*59 affects the distribution of HSPB8 WT and of other CASA complex components in NSC34 cells transiently transfected with fluorescently tagged HSPB8 WT, BAG3, or HSPA1A. Cells transfected with an empty vector (EV) or untagged HSPB8 WT showed a diffuse distribution of GFP-HSPB8 WT, GFP-BAG3, and YFP-HSPA1A (Fig. 3E). Instead, in the presence of HSPB8 p.Q188Rfs*59, puncta containing the HSPB8 mutant were also positive for GFP-HSPB8 WT or GFP-BAG3 or YFP- HSPA1A, indicating sequestration of these proteins into aggregates of the mutant protein. These results align with our findings on the HSPB8 frameshift mutants with the shorter highly hydrophobic C-terminus [16]: despite different length and sequence, the new C-terminal extension from HSPB8 frameshift p.Q188Rfs*59, p.Y174Qfs*72, and p.P172Lfs*75 does not alter the HSPB8 capability to interact with other CASA complex members, but induces their co-aggregation.

### Proteostasis is impaired by the novel HSPB8 frameshift mutations

**Fig. 4:**
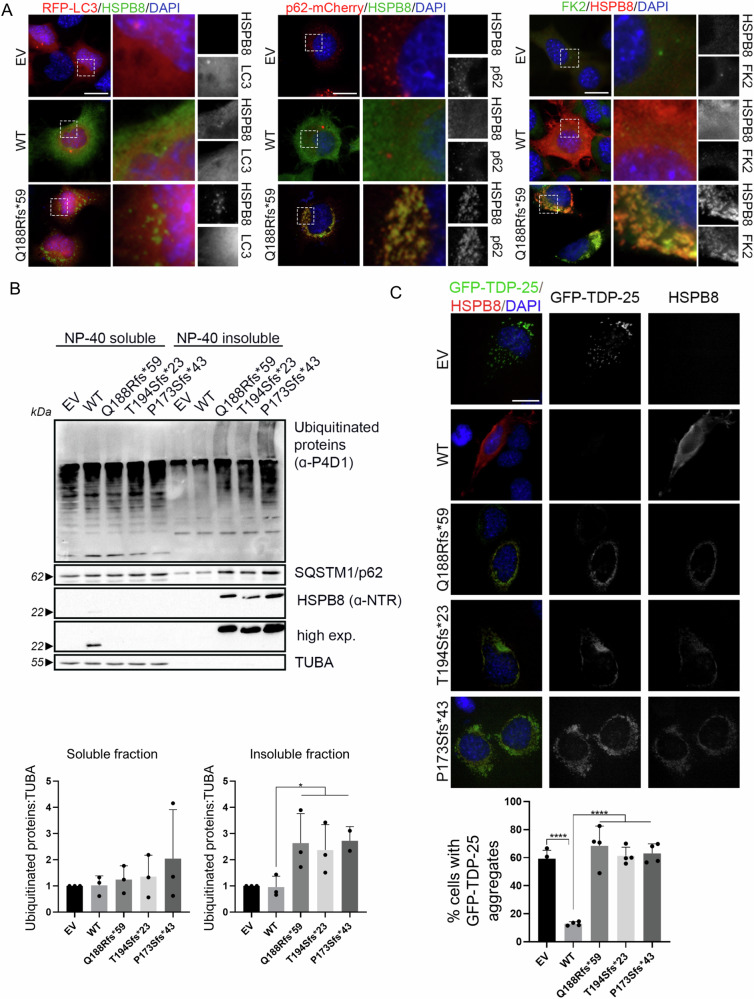
Proteostasis is impaired by the novel HSPB8 frameshift variants. **A** Immunofluorescence on NSC34 transiently transfected with an empty vector (EV), HSPB8 WT or its mutant p.Q188Rfs*59 (representative of the novel frameshift variants) and RFP-tagged LC3 (left, red) or mCherry-tagged SQSTM1/p62 (indicated as p62, middle, red) or ubiquitinated proteins (FK2, right, green). Nuclei are stained with DAPI (blue); scale bar = 20 µm. **B** Western blot on NP-40 soluble and insoluble protein fractions of NSC34 transiently expressing HSPB8 WT or its mutants p.Q188Rfs*59, p.T194Sfs*23, or p.P173Sfs*43. HSPB8 was detected using an antibody recognizing the N-terminal region (NTR). Bar graphs report mean values (±SD) of densitometry of ubiquitinated proteins on soluble tubulin (TUBA), normalised to control cells (EV). One-tailed unpaired student’s *t* test was performed (*p < 0.05); *N* = 3. **D** Immunofluorescence on NSC34 transiently transfected with an empty vector (EV), HSPB8 WT or its mutants p.Q188Rfs*59, p.T194Sfs*23 or p.P173Sfs*43 (red) and GFP-TDP-25 (green). Nuclei are stained with DAPI (blue); scale bar = 20 µm. Bar graph reports the percentage of cells with GFP-TDP-25 aggregates. Five random fields from four samples were analysed. Cells expressing GFP-TDP-25 were manually counted with a total number of cells *n* = 223 (EV), 216 (WT), 185 (p.Q188Rfs*59), 199 (p.T194Sfs*23), 224 (p.P173Sfs*43). One-way ANOVA with Tukey’s test was performed (****p < 0.0001); *n* = 4.

Next, we assessed if the new HSPB8 C-terminal extension alters proteostasis. We evaluated the effect of HSPB8 WT and mutant on autophagy using the autophagy markers RFP-tagged LC3 and mCherry-tagged SQSTM1/p62 (Fig. 4A). By fluorescence microscopy analysis, we observed that neither HSPB8 WT nor p.Q188R*fs59 altered RFP-LC3 distribution, which remained substantially diffuse. Instead, mCherry-SQSTM1/p62 displayed a characteristic punctate distribution both in cells expressing the empty vector and HSPB8 WT, while it was fully entrapped within HSPB8 mutant aggregates in HSPB8 p.Q188Rfs*59 expressing cells. These aggregates were also positive for ubiquitinated proteins (Fig. 4A). In agreement, SQSTM1/p62 and ubiquitinated proteins were enriched in NP-40 insoluble fractions analysed in western blot, likewise in cells expressing HSPB8 p.T194Sfs*23 and p.P173Sfs*43 mutants (Fig. 4B). Therefore, we tested if HSPB8 p.Q188Rfs*59 undermines CASA activity, by using an aggregation assay to assess the ability of this HSPB8 mutant to counteract the aggregation propensity of a typical CASA substrate, the aggregation-prone 25 kDa fragment of TDP-43 (known as TDP-25). By fluorescent microscopy, we observed that HSPB8 p.Q188Rfs*59 co-aggregated with a GFP-tagged TDP-25 comparably to the known HSPB8 frameshift mutants (Fig. 4C), as we previously reported [16]. Our results demonstrated that the novel C-terminal extension in HSPB8 severely affects proteostasis, similarly to the alternative C-terminal extension already studied.

## Discussion

We identified three novel pathogenic variants - c.562delC, c.520_523delTACT, and c.515delC - in the *HSPB8* gene in three patients with early-onset myopathy, with severe respiratory involvement and, possibly, cardiac dysfunction. Until now, missense and frameshift *HSPB8* mutations have been associated with hereditary (mostly motor) neuropathies, neuromyopathy, and pure myopathy, mainly of adult-onset [8, 9, 11, 15], but not with cardiomyopathy, suggesting that the novel frameshift mutations may display other mechanisms of pathogenicity that affect various striated muscles. Patient I phenotype resembles a neuromyopathy with predominantly distal involvement of leg muscles; it started in his early twenties with progressive disease course, later affecting proximal leg muscles, upper extremities muscles, and axial muscles, with rapid weakness progression, and severe affection of respiratory and cardiac muscles leading to premature death in his 30s. Patient II phenotype was pure myopathy, with predominant weakness affecting truncal, abdominal, and proximal lower limb muscles. Patient II developed Kawasaki syndrome in early childhood, followed by cardiomyopathy at 4 years of age, with long-term complications primarily affecting the heart and persistent coronary artery aneurysms. This may lead to thrombosis, stenosis, or myocardial infarction, with risk for coronary artery stenosis, chronic myocardial ischemia, arrhythmias, and eventual heart failure due to recurrent ischemic damage [26], as well as to valvular heart disease, increasing risks of early-onset atherosclerosis from lasting vascular changes [27]. Although cardiomyopathy is infrequent in Kawasaki disease, some cases have involved dilated cardiomyopathy [28]. Conversely, patient II developed restrictive hypertrophic cardiomyopathy similar to patient I. Patient III displays a pure myopathy phenotype, with proximal, distal, and axial muscle weakness, restricted lung defect, left elevated hemidiaphragm, and severe scoliosis. Patient III exhibited respiratory insufficiency as patient I and patient II, but no signs of cardiomyopathy at evaluation. The family history revealed a familial inheritance of the *HSPB8* variant, with his mother exhibiting similar, but milder, phenotypes and his maternal uncle developing myopathy and passing away from cardiomyopathy in his 50s.

So far, only a few patients with *HSPB8* variants showed respiratory insufficiency, and none of them had cardiomyopathy [12, 14], which we found in our patients I and II, and the uncle of patient III. Although cardiomyopathy might be unrelated to *HSPB8*, this chance is small, as WES analysis excluded relevant variants in cardiomyopathy-associated genes. Notably, mice studies correlated HSPB8 with cardiac functionality: transgenic mice overexpressing mutant HSPB8 in cardiac tissue exhibited mild cardiomyopathy [29], while no obvious phenotype was observed in an HSPB8 knock-out mouse [30]. Cardiac functional and structural alterations with ageing or upon cardiac overload were described in another knock-out mouse model [31, 32]. Noteworthy, mutations in *BAG3*, the obligatory partner of HSPB8, cause cardiomyopathy, besides neuropathy and myopathy [33–36], and mutations in other HSPBs (HSPB5, HSPB6, and HSPB7) also associate with cardiac pathology [37].

Patients’ muscle biopsy showed muscular dystrophy patterns with inclusions and rimmed vacuoles, consistent with findings in HSPB8-associated (neuro)myopathy [9]. The muscle MRI findings were indicative of diffuse pathology in leg muscles with relative sparing of the posterior compartment of the lower legs consistent with cases of HSPB8 pathology [38].

At the molecular level, the new HSPB8 frameshift variants have a C-terminal extension translated from the ORF alternative from that of other HSPB8 frameshift variants previously identified. However, the pathogenic mechanisms related to the HSPB8 p.Q188Rfs*59, p.Y174Qfs*72, and p.P172Lfs*75 variants are comparable to those reported in other known variants with a C-terminal extension that strongly reduces HSPB8 solubility [16], causing their aggregation and the sequestration of HSPB8 WT and other CASA complex components. Importantly, all HSPB8 frameshift mutations described so far have a C-terminus highly enriched with hydrophobic amino acids, sufficient to drive HSPB8 aggregation and proteostasis dysfunction. Instead, the features and mechanisms by which the novel C-terminal extension affects HSPB8 aggregation are still unclear. Nevertheless, overexpression of the representative HSPB8 p.Q188Rfs*59 caused CASA and proteostasis defects, recapitulating the data we previously obtained for the other HSPB8 frameshift mutations [16]. Notably, with regard to the known HSPB8 frameshift mutants with the shorter C-terminal extension (see Fig. 3A), there is no evidence of HSPB8 aggregation in the muscle of patients. Indeed, the elongated protein has not been detected in a muscle biopsy of a patient with the Q170Gfs*45 [15], and fibroblasts of patients with the HSPB8 p.P173Sfs*43 variant have reduced HSPB8 expression, suggesting that mRNA decay, or, more likely, protein degradation, might be involved in mutant decreased level [12]. Conversely, one of the novel HSPB8 frameshift mutants with the longer C-terminal extension clearly aggregated in muscle specimens of an affected patient [24]. Whether cells respond differently to the two different C-terminal extensions is still unknown. It is interesting to note that the patients we described here show an early and more aggressive disease with cardiomyopathy.

Therefore, our findings significantly expand the genotypic and phenotypic spectra of *HSPB8*-related diseases and emphasize the relevant role of HSPB8 in cardiac tissue, like in skeletal muscle and neurons. Moreover, these new HSPB8 C-terminal variants reveal that the last *HSPB8* exon is highly susceptible to mutations that are likely pathogenic because of the intrinsic nature of the 3’-UTR. Our study underlines the importance of *HSPB8* genetic testing in neuropathy and (cardio)myopathy in undiagnosed patients, even in the absence of clear autosomal dominant inheritance pattern in a family.

## Supplementary information

Supplementary material

## Data Availability

The data that support the findings of this study are available from the corresponding author, upon reasonable request.
