# Supplementary material for: Novel HSPB8 mutations in severe early-onset myopathy with involvement of respiratory and cardiac muscles cause proteostasis defects in cell models
Source: Eur J Hum Genet. 2025 Jun 4;33(8):1015–24. doi: 10.1038/s41431-025-01868-z (PMC12322164; doi:10.1038/s41431-025-01868-z)
Supplement: Supplementary file 1 — Supplementary material [file 41431_2025_1868_MOESM1_ESM.pdf]

Title: Novel HSPB8 mutations in severe early-onset myopathy with involvement of respiratory and cardiac muscles cause proteostasis defects in cell models.

Barbara Tedesco,[*] Stojan Peric,[*] Goknur Selen Kocak[#], Jiayan Tan[#], Han Duong[#], Ana Töpf, Vidosava Rakocevic-Stojanovic, Sanja Milenkovic, Yolande Parkhurst, Liliane Gibbs, Angela Martin-Rios, Pier D. Lambiase, Oliver P. Guttmann, Chiara Marini-Bettolo, Elizabeth Harris, Matthew B. Harms, Vukan Ivanovic, Veronica Marchesi, Margherita Milone, Vincent Timmerman, Volker Straub, Angelo Poletti,[°] Virginia Kimonis,[°]

[*] Co-first and [°]Co-Last authors; [#]These authors contributed equally to this work.

Supplementary Material

Patient recruitment and Ethical Approval

Patient I was recruited at the Neurology Clinic, University Clinical Centre of Serbia, Faculty of Medicine, University of Belgrade, Serbia. Patient II received care at the Highly Specialised Service for Rare Neuromuscular Disorders in Newcastle upon Tyne, UK. Patient III was recruited at University of California Irvine, USA.

Ethical approval was granted by the Newcastle and North Tyneside Research Ethics Committee (REC reference number 09/H0906/28), the Faculty of Medicine, University of Belgrade, and the University of California Irvine Institutional Review Board (IRB 2007-5832). Appropriate informed written consent, including publication of anonymized data, was provided.

Protein solubility prediction

CamSol web-server (http://www-vendruscolo.ch.cam.ac.uk/camsolmethod.html) was used to obtain the intrinsic solubility profile of HSPB8 wild-type and variants. Starting from aminoacid Ala168, the HSPB8 protein sequence (NP_055180.1) or the predicted sequence of the variants were used as inputs, following the procedure described in (1).

Cell culture and transfection

NSC34 (kindly provided by Dr. Neil Cashman, University of Toronto, Toronto, ON, Canada) were grown in high glucose DMEM (EuroClone, ECB7501L; Pero, MI, Italy), added with glutamine 1 mM (EuroClone, ECB3004D), penicillin/streptomycin (EuroClone, ECB3001D) and 5% fetal bovine serum (FBS, Sigma-Aldrich, F7524; St. Louis, MO, USA) at 37°C, 5% CO_2. The day after seeding, cells were transfected with Lipofectamine3000/P3000 reagent (Invitrogen, L3000001). The plasmids used are listed in **Supplementary Table 3**. PCi-HSPB8_fs p.Q188Rfs*59 was obtained following the subcloning strategy previously described (2).

Protein extraction and western blot

Forty-eight hours after transfection, cells were harvested by centrifugation for 5 min at 100 g at 4°C and lysed using NP-40 lysis buffer (150 mM NaCl [Sigma-Aldrich, S3014], 20 mM TrisBase [Sigma-Aldrich, T1503], Nonidet P-40 0.5% [NP-40; Sigma-Aldrich, 98379], 1.5 mM $MgCl_2$, glycerol 3% [Sigma-Aldrich, G5516], pH 7.4) with protease inhibitors cocktail (Sigma-Aldrich, P8340) and 1 mM DTT (Merck Millipore, 11474). NP-40 soluble/insoluble protein extraction, SDS-PAGE and western blot were performed as previously described (2). Western blot membranes were incubated with a blocking solution of 5% non-fat dried milk (BioBasic, Toronto, Canada, NB0669) in TBS-Tween (20 mM TrisBase [Sigma-Aldrich, T1503], 140 mM NaCl [Sigma-Aldrich, S3014], pH 7.6 and 0.01% Tween 20 [Sigma-Aldrich, P1379]) for 1 h and then incubated with primary antibodies diluted in the same solution overnight. Three washing steps were made with TBS-Tween for 10 min and secondary antibodies were added. Antibodies are listed in **Supplementary Table 2**. Enhanced chemiluminescent (ECL) detection reagent (Cyanagen Reagents for Molecular Biology, ECL Westar Antares XLS142; Bologna, Italy,) was used for detection and images were acquired using a Chemidoc XRS System (Bio-Rad Laboratories, California, USA). Optical densities of the bands were analyzed using Image Lab Software (Bio-Rad Laboratories).

Fluorescence microscopy

Cells were fixed with a solution of 4% paraformaldehyde in 0.2 M phosphate buffer (0.06 M KH_2PO_4, 0.31 M Na_2HPO_4, pH 7.4) for 25 min at 37°C and then washed with PBS (Sigma-Aldrich, P4417) solution 3 times for 5 min. Permeabilization and blocking were performed using a solution of 0.1% Triton X-100 (Sigma-Aldrich, X100), 1% bovine serum albumin (BSA) (Sigma-Aldrich, A9418), and 10% FBS (Gibco, 10270106) in PBS for 40 minutes at room temperature (RT). Then, the primary antibodies diluted in 0.1% BSA in PBS were incubated overnight at 4°C. The following day, cells were washed with PBS, and secondary antibodies diluted in 0.1% BSA in PBS were incubated for 1 h at RT. Three washing steps were made with PBS, with the middle one containing DAPI (Sigma-Aldrich, D9542) to stain nuclei. MOWIOL mounting medium was used to mount the coverslips onto slides. Confocal microscopy images were captured using a Zeiss LSM900 confocal microscope and

analyzed with ImageJ/Fiji. Fluorescence microscopy was performed with an Axiovert 200 microscope (Zeiss) with a photometric CoolSnap CCD camera (Robber Scientific) using the Metamorph software (Universal Imaging). Antibodies are listed in **Supplementary Table 2**.

Statistics

One-Way ANOVA with Tukey's multiple comparisons test or one-tailed unpaired Student's t test were performed as indicated using PRISM software (GraphPad Software).

Supplementary Figure 1. Sequencing trace files of families 1 and 2. A. Sequencing chromatograms of *HSPB8* gene in family 1. B. Sequencing results of *HSPB8* gene in family 2. C. Sequencing chromatograms of *HSPB8* gene in patient III.

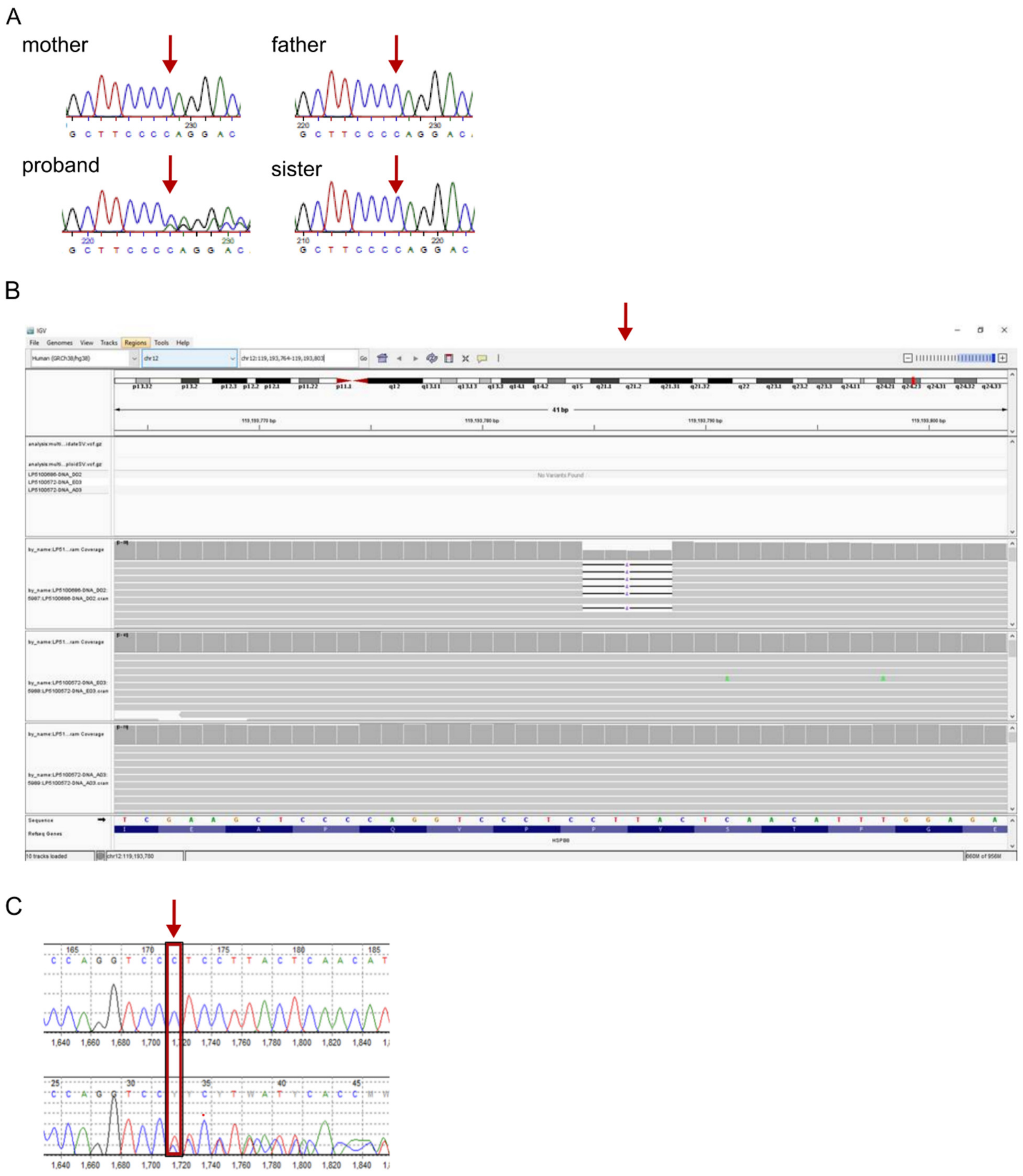

Supplementary table 1. Reported cases of HSPB8 frameshift mutations carriers and clinical manifestations. DLL = distal lower limbs; UL = upper limbs; LL = lower limbs; PLL = proximal lower limbs; M = male, F = female; NA = not assessed; RV = rimmed vacuoles.

FAMILY	INDIVIDUAL	AGE OF ONSET (Y.)	WEAKNESS AT ONSET	SEX	PULMONARY FUNCTION	RESPIRATORY INVOLVEMENT	NEURONAL INVOLVEMENT	CARDIAC INVOLVEMENT	MUSCLE HISTOLOGY	HSPB8 VARIANT	REFERENCE
**1**	III-2	46	DLL	M	NA	-	+	-	Dystrophic features, RV, myofibrillar pathology, denervation atrophy	c.515dupC, p.Pro173Serfs*43	(3)
**2**	II-2	40	DLL	M	NA	-	-	-	Dystrophic features, RV, myofibrillar pathology	c.508_509delCA, p.Gln170Glyfs*45	(4)
	II-8	35	DLL	M	NA	-	-	-	Dystrophic features, RV, myofibrillar pathology	c.508_509delCA, p.Gln170Glyfs*45	(4)
	III-2	40	DLL	F	NA	-	-	-	Dystrophic features, RV, myofibrillar pathology	c.508_509delCA, p.Gln170Glyfs*45	(4)
**3**	III-1	40	DLL	M	NA	-	-	-	Dystrophic features, RV, myofibrillar pathology	c.508_509delCA, p.Gln170Glyfs*45	(4)
**4**	II-1	40	DLL	F	NA	-	-	-	Dystrophic features, RV, myofibrillar pathology	c.508_509delCA, p.Gln170Glyfs*45	(4)
**5**	IV-8	35	DLL	M	+	+	-	NA	Dystrophic features, RV	c.515dupC, p.Pro173Serfs*43	(5)
	III-14	56	UL+ LL	F	-	-	-	NA	Dystrophic features, RV	c.515dupC, p.Pro173Serfs*43	(5)
	III-15	42	DLL	M	+	+	+	-	Dystrophic features, RV	c.515dupC, p.Pro173Serfs*43	(5)
**6**	1	19	PLL	M	-	-	-	-	RV, myofibrillar pathology	c.577_580dupGTCA, p.Thr194Serfs*23	(6)
**7**	III-5	37		F	-	-	-	-		c.525_529delAACAT, p.Thr176Trpfs*38	(7)
	III-1	Late 20s		M	NA	+	NA	-	RV, myofibrillar pathology	c.525_529delAACAT, p.Thr176Trpfs*38	(7)
	II-3	Late 40s		M	NA	+	NA	-		c.525_529delAACAT, p.Thr176Trpfs*38	(7)
	III-4	40		M	NA	-	NA	-		c.525_529delAACAT, p.Thr176Trpfs*38	(7)
	II-10	50		F	+	+	NA	-		c.525_529delAACAT, p.Thr176Trpfs*38	(7)
	III-9	35		F	+	+/-	NA	-		c.525_529delAACAT, p.Thr176Trpfs*38	(7)
**8**	II-5	6	PLL	F		+	-	-	RV, myofibrillar pathology	c.576_579delinsCAG p.Glu192Aspfs*55	(8)
**9**	1	Before 20	DLL	M	+	+	+	+	Dystrophic features, RV, myofibrillar pathology	c.562delC, p.Gln188Argfs*59	This report
**10**	1	Before 10	PLL	F	+	+	-	+	Dystrophic features, RV, myofibrillar pathology	c.520_523delTACT, p.Tyr174Glnfs*72	This report

**11**	III-1	30	PL	M	+	+	-	-	Dystrophic features, RV, myofibrillar pathology	c.515delC, p.Pro172Leufs*75	This report
	II-2	40s-50s	LL	F	NA	NA	NA	-	Myofibrillar disruption	c.515delC, p.Pro172Leufs*75	This report

Supplementary table 2. List of antibodies used in this study**.**

Antibody	Host species	Dilution			Source
		IHC	WB	IF	
Myosin fast-twitch (Type II) heavy-chain Ab2 (Clone MYSN02)	Mouse monoclonal	1:500 – 1:1,000 (Patient 1)			Thermo scientific, MS-1236-R7
Myosin heavy chain (Fast) (Clone WB-MHCF)	Mouse monoclonal	1:40 (Patient 2)			Leica Biosystems, NCL-MHCf
Desmin Clone D33	Mouse monoclonal	1:100 (Patient 1)			Dako, M0760
Desmin (Clone D33)	Mouse monoclonal	1:400 (Patient 2)			Agilent, D33
HSPB8	Rabbit monoclonal	1:200 (Patient 1)	1:1,000	1:500	Invitrogen, MA5-32421 (JJ08-53)
HSPB8 (Clone OTI1E3)	Mouse monoclonal	1:50 (Patient 2)			Thermo Fisher Scientific, MA5-25112
HSPB8	Mouse monoclonal			1:250	R&D systems, MAB-4987
TARDBP/TDP-43	Rabbit monoclonal	1:100 (Patient 1)			Abcam, ab190963
TARDBP/TDP-43 (Clone 2E2-D3)	Mouse monoclonal	1:800 (Patient 2)			Novus Bio, H00023435

TUBA4A/ α-tubulin	Mouse monoclonal		1:2,000		Sigma-Aldrich, T6199
Ubiquitin	Mouse monoclonal		1:500		Santa Cruz Biotechnology, sc-8017
Ubiquitinated proteins (FK2)	Mouse monoclonal			1:500	Merck Millipore, 04–263
SQSTM1/p62	Rabbit polyclonal	1:500 (Patient 1)			Abcam, ab233207
SQSTM1/p62	Mouse monoclonal	1:150 (Patient 2)			Abcam, ab56416
SQSTM1/p62	Rabbit polyclonal		1:2,000		Sigma-Aldrich, P0067
GFP	Mouse monoclonal		1:1,000		Immunological Sciences, MAB-94345
**II antibody**					
anti-mouse HRP	rabbit	1:100			Agilent, P026002-2
anti-rabbit IgG-HRP	goat		1:10,000		Jackson ImmunoResearch Laboratories, Inc., 111–035-003
anti-mouse IgG-HRP	goat		1:10,000		Jackson ImmunoResearch Laboratories, Inc., 115–035-003

anti-rabbit 594 Alexa Fluor	goat			1:1,000	Life Technologies, Thermo Fisher, A-11012
anti-mouse 647 Alexa Fluor	goat			1:1,000	Life Technologies, Thermo Fisher, A-21235
anti-rabbit 488 Alexa Fluor	goat			1:1,000	Life Technologies, Thermo Fisher, A-11008
anti-mouse 488 Alexa Fluor	goat			1:1,000	Life Technologies, Thermo Fisher, A-11017

Supplementary table 3. List of plasmids used in this study.

Plasmid		Source	Reference
pCDNA3	Empty vector (EV)	Invitrogen, V790-20	
pCi-HSPB8_WT	HSPB8 WT		(9)
pCi-HSPB8 p.P173Sfs*43	HSPB8 frameshift mutant p.P173Sfs*43		(2)
pCi-HSPB8 p.T194Sfs*23	HSPB8 frameshift mutant p.T194Sfs*23		(2)
pCi-HSPB8 p.Q188Rfs*59	HSPB8 frameshift mutant p.Q188Rfs*59	This study	
p.HSPB8 GFP WT	GFP-tagged HSPB8 WT		(2)
pBAG3-GFP WT	GFP-tagged BAG3 WT	Dr. Josée N. Lavoie (Université Laval, Québec, Canada)	(10)

pEYFP-N1-Hsp70	YFP-tagged HSP70 (HSPA1A/B)	Dr. Richard Morimoto (Department of Molecular Biosciences, Rice Institute for Biomedical Research, Northwestern University, Evanston, IL, USA)	(11)
pDEST-mCherry-p62	mCherry-tagged SQSTM1	Dr. Terje Johansen (The Artic University of Norway, Norway)	(12)
pmRFP-LC3	RFP-tagged LC3	Dr. Tamotsu Yoshimori (Addgene plasmid # 21075)	(13)
pEGFP-TDP-25	GFP-tagged TARDBP/TDP-43 C-terminal fragment (25 kDa)	Dr. Leonard Petrucelli (Department of Neuroscience, Mayo Clinic, Jacksonville, FL 32224, USA)	(14)

References

1. Sormanni P, Aprile FA, Vendruscolo M. The CamSol method of rational design of protein mutants with enhanced solubility. J Mol Biol [Internet]. 2015;427(2):478–90. Available from: https://www.ncbi.nlm.nih.gov/pubmed/25451785

2. Tedesco B, Vendredy L, Adriaenssens E, Cozzi M, Asselbergh B, Crippa V, et al. HSPB8 frameshift mutant aggregates weaken chaperone-assisted selective autophagy in neuromyopathies. Autophagy. 2023 Feb 28;1–23.

3. Ghaoui R, Palmio J, Brewer J, Lek M, Needham M, Evilä A, et al. Mutations in HSPB8 causing a new phenotype of distal myopathy and motor neuropathy. Neurology. 2016;86(4):391–8.

4. Echaniz-Laguna A, Lornage X, Lannes B, Schneider R, Bierry G, Dondaine N, et al. HSPB8 haploinsufficiency causes dominant adult-onset axial and distal myopathy. Acta Neuropathol. 2017;134(1):163–5.

5. Al-Tahan S, Weiss L, Yu H, Tang S, Saporta M, Vihola A, et al. New family with HSPB8-associated autosomal dominant rimmed vacuolar myopathy. Neurol Genet. 2019 Aug;5(4):e349.

6. Nicolau S, Liewluck T, Elliott JL, Engel AG, Milone M. A novel heterozygous mutation in the C-terminal region of HSPB8 leads to limb-girdle rimmed vacuolar myopathy. Neuromuscul Disord. 2020;

7. Inoue-Shibui A, Niihori T, Kobayashi M, Suzuki N, Izumi R, Warita H, et al. A novel deletion in the C-terminal region of HSPB8 in a family with rimmed vacuolar myopathy. J Hum Genet. 2021 Oct;66(10):965–72.

8. Yang G, Lv X, Yang M, Feng Y, Wang G, Yan C, et al. Expanding the spectrum of HSPB8-related myopathy: a novel mutation causing atypical pediatric-onset axial and limb-girdle

involvement with autophagy abnormalities and molecular dynamics studies. J Hum Genet. 2024 Nov 15;

9. Crippa V, Sau D, Rusmini P, Boncoraglio A, Onesto E, Bolzoni E, et al. The small heat shock protein B8 (HspB8) promotes autophagic removal of misfolded proteins involved in amyotrophic lateral sclerosis (ALS). Hum Mol Genet. 2010;19(17):3440–56.

10. Fuchs M, Luthold C, Guilbert SM, Varlet AA, Lambert H, Jetté A, et al. A Role for the Chaperone Complex BAG3-HSPB8 in Actin Dynamics, Spindle Orientation and Proper Chromosome Segregation during Mitosis. PLoS Genet. 2015;11(10):e1005582.

11. Kim S, Nollen EAA, Kitagawa K, Bindokas VP, Morimoto RI. Polyglutamine protein aggregates are dynamic. Nat Cell Biol. 2002 Oct 30;4(10):826–31.

12. Pankiv S, Clausen TH, Lamark T, Brech A, Bruun JA, Outzen H, et al. p62/SQSTM1 Binds Directly to Atg8/LC3 to Facilitate Degradation of Ubiquitinated Protein Aggregates by Autophagy. Journal of Biological Chemistry. 2007 Aug;282(33):24131–45.

13. Kimura S, Noda T, Yoshimori T. Dissection of the autophagosome maturation process by a novel reporter protein, tandem fluorescent-tagged LC3. Autophagy. 2007;3(5):452–60.

14. Zhang YJ, Xu YF, Cook C, Gendron TF, Roettges P, Link CD, et al. Aberrant cleavage of TDP-43 enhances aggregation and cellular toxicity. Proc Natl Acad Sci U S A. 2009 May 5;106(18):7607–12.
